# Minimally invasive management of lower midline diastema: A case report and literature review

**DOI:** 10.1097/MD.0000000000048618

**Published:** 2026-05-01

**Authors:** Ghadah Khaled Bahamdan, Norah Mohammed Alshehri, Faisal Hasan Alshehri

**Affiliations:** a Department of Periodontics and Community Dental Sciences, College of Dentistry, King Khalid University, Abha, Saudi Arabia; b Department of Restorative Dentistry, Faculty of Dentistry, King Abdulaziz University, Jeddah, Saudi Arabia; c Department of Restorative Dental Science, College of Dentistry, King Khalid University, Abha, Saudi Arabia.

**Keywords:** free gingival graft, midline diastema, minimally invasive, resin-bonded bridge

## Abstract

**Rationale:**

Midline diastema is an unpleasant condition that affects a patient’s smile and causes significant aesthetic concerns. Treating diastema and achieving the desired aesthetics, function, and harmony can be challenging without invasive treatment. This case report demonstrates a minimally invasive approach to closing a lower midline diastema.

**Patient concerns:**

A 38-year-old female patient presented to the dental clinic at King Khalid University with the primary complaint of a “large space in the lower front tooth region.” Her medical history indicated no significant health concerns.

**Diagnoses:**

The patient was diagnosed with localized periodontitis, stage III, grade B. She presented with a Miller class III gingival recession at the lower central incisors, an aberrant frenum, a decreased width of keratinized gingiva, and a midline diastema of 6 mm.

**Interventions:**

A free gingival graft is used to increase the width of the keratinized gingiva, followed by the placement of a zirconia resin-bonded bridge to close the space.

**Outcomes:**

The treatment yielded a satisfactory outcome, as evidenced by an increase in the width of keratinized gingiva from 1 mm at baseline to 5 mm at 4 months and a gain of 1 to 2 mm in clinical attachment level. Two months following surgery, complete closure of a 6-mm lower midline diastema was successfully achieved using a zirconia resin-bonded bridge.

**Lessons:**

The use of a free gingival graft effectively increases the width of the keratinized gingiva. A resin-bonded bridge offers satisfactory aesthetics and functionality, providing a conservative treatment that makes it a favorable option for closing lower midline diastema compared with more invasive and time-consuming procedures, such as bone grafting or orthodontic treatment.

## 1. Introduction

Midline diastema is “midline anterior spacing exceeding 0.5 mm between adjacent anterior teeth.”^[1]^ This is an unpleasant condition that affects the patient’s smile and causes significant aesthetic concerns for both the patient and observers.^[2]^ According to epidemiological research, midline diastema is quite widespread in children, ranging from 43% to 97%. It declines drastically between the ages of 9 and 11, and then gradually diminishes until the age of 15.^[3,4]^

Diastema can be caused by an enlarged labial frenum, a midline bony cleft, or other factors such as dentoalveolar diastemas associated with normal growth and development, pernicious habits, soft tissue or muscular imbalances, physical impediments, abnormal maxillary arch structure, dental anomalies, and other malocclusions.^[2–5]^

An enlarged maxillary labial frenum has been shown to cause anterior diastema in maxillary teeth.^[3,5–8]^ The principal cause of diastemas in mandibular teeth is tongue thrust at a low rest posture.^[9]^ Diastema has to be identified through a detailed medical and dental history, a meticulous clinical examination, and radiographic assessment. In addition, diagnostic study models are essential for measurement and analysis, particularly when a diastema arises from malocclusion or discrepancies in tooth and/or arch dimensions.^[2–4]^

According to contemporary research, diastema may be treated with restorative, orthodontic, periodontal, or a combination of these treatments.^[2,10]^ Orthodontic therapy is beneficial for treating diastemas; nevertheless, it is expensive, takes longer to complete, and does not always provide desirable outcomes.^[11–13]^

As a result, restorative therapy, with or without periodontal surgery, is regarded as the most effective therapeutic approach. This can be achieved via composite restorations, direct resin application, or indirect porcelain laminate veneers, all of which are less invasive procedures.^[13–17]^ Periodontal surgical therapy, such as gingivectomy, gingival grafting, crown lengthening, soft tissue repositioning, or frenectomy, is sometimes necessary to achieve optimal soft tissue form and health.^[11,18,19]^

Diastema may affect a patient’s smile and cause significant aesthetic concerns for the patient and the spectator. In certain circumstances, closing diastemas, especially with large spaces, is a complex therapy technique. Treating diastema and achieving the desired aesthetics, function, and harmony can be challenging without multidisciplinary participation, which may involve orthodontics with or without periodontal intervention.

Due to recent advancements in treatment options and dental technology, dentists have access to a wide range of equipment and treatment alternatives. The final notion of “minimally invasive treatment approaches” yields excellent outcomes, providing functional, aesthetically pleasing, and restorative benefits over a lifetime.

Gingival recession refers to the apical displacement of the gingival margin toward the cementoenamel junction.^[20]^ Numerous factors contribute to gingival recession, including plaque-induced inflammation, trauma from toothbrushing, tooth malposition, alveolar bone dehiscence, iatrogenic factors such as restorative or orthodontic procedures, high muscle attachment, aberrant frenum attachment, occlusal injury, and other contributing factors.^[20,21]^ Periodontal therapy is necessary when the recession is severe and the gingival health cannot be maintained. Treatment of mucogingival conditions is critical for preventing further gingival recession and improving oral plaque control.^[20,22,23]^

Mandibular incisor teeth have a reduced amount of attached gingiva on their labial surface, which may increase their susceptibility to periodontal disease.^[24]^ Various mucogingival surgical procedures are employed, including gingival grafting, frenectomy, or a combination of both.^[25]^ In situations with apparent labial frenum attachment that causes a gingival recession, a frenectomy is suggested.^[26]^

A frenectomy procedure can be combined with a free gingival graft (FGG) to deepen the vestibule, widen the attached gingiva, and prevent recurrence of the labial frenum.^[25,27]^ FGG is utilized to increase the width of the attached gingiva. This approach is highly effective in increasing keratinized and attached gingiva while halting further gingival recession.^[28–30]^

In certain circumstances, when a midline diastema is present, patients may refuse orthodontic treatment; therefore, alternative procedures, such as resin-bonded bridges (RBBs; also known as resin-bonded fixed partial dentures [RBFPDs]), may be a viable option.^[31]^ RBFPDs were introduced in dentistry in the 1970s, and Rochette was the first to describe them.^[32,33]^ RBFPDs need little to no tooth preparation.^[33]^ A high success rate has been reported, motivating dentists to utilize it in the posterior region.^[34,35]^

The material from which the resin-bonded bridge is fabricated might be metallic or nonmetallic. Recent systematic literature analysis has revealed a diverse range of materials for RBFPDs, including metal-ceramic, metal-resin, composite, glass-infiltrated ceramic, reinforced-glass ceramic, and densely sintered zirconia. The latter has shown superior 5-year survival rates compared with the other materials.^[36]^ To address the patient’s aesthetic concerns, tooth-colored materials such as zirconium oxide ^[37–40]^ and fiber-reinforced composites can be regarded as the materials of choice.^[41–44]^ They may be used as the framework for resin-bonded prostheses. Clinicians have replaced metal frameworks with RBFPDs, traditionally known as Maryland bridges, using materials such as zirconia, glass ceramic, fiber-reinforced composite, or lithium disilicate, which have long-term evidence supporting their use.^[39,44–49]^

This case report discusses the multidisciplinary treatment of a patient with a large midline diastema, deep gingival recession, and insufficient attached and keratinized gingiva in the anterior mandibular teeth. The patient sought treatment primarily for aesthetic concerns regarding the closure of the diastema. This article uniquely illustrates a combined FGG and zirconia RBFPD approach in the treatment of lower midline diastema, where insufficient keratinized tissue and apparent frenulum attachment often hinder long-term stability.

The primary objective was to enhance the mucogingival condition, followed by closing the diastema using an RBFPD.

## 2. Case presentation

A 38-year-old female patient presented to the dental clinic at King Khalid University Hospital with the primary complaint of a “large space in the lower front tooth region.” Her medical history indicated no significant health concerns. Her dental history revealed that her last visit to a dentist was 7 years ago, during which she received restorations. The patient’s oral hygiene was found to be inadequate, with localized calculus observed around the lower anterior teeth and a report of mild halitosis.

An intraoral and periodontal examination was performed, including clinical parameters such as plaque control record, bleeding on probing (BOP), probing depth, clinical attachment level (CAL), and recession depth (the distance between the cementoenamel junction and the gingival margin). At baseline, probing depth ranged from 2 to 3 mm, CAL from 4 to 7 mm, and recession depth from 2 to 6 mm. Clinical findings showed generalized BOP, gingival inflammation, and gingival recession. Miller class III gingival recession was noted on teeth #31 and #41 (Fig. 1).^[50]^ In addition, the patient presented with an aberrant frenum, decreased width of keratinized gingiva about 1 mm, inadequate vestibular depth, spacing (a midline diastema of 6 mm), and tilting of teeth without mobility.

**Figure 1.**
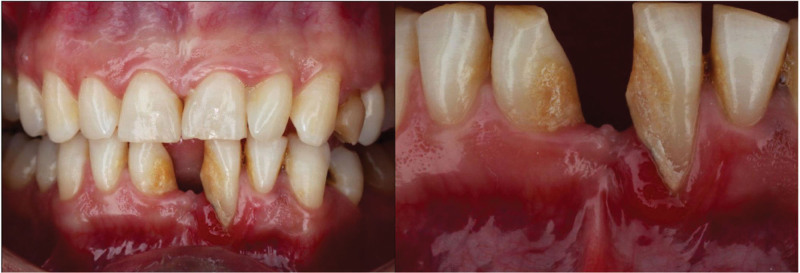
Preoperative intraoral photograph showing a 6-mm midline diastema between the mandibular central incisors (#31 and #41). Gingival recession and inadequate width of keratinized gingiva are evident in the region planned for grafting.

Intraoral periapical radiographs revealed horizontal bone loss extending to the middle third of the root (Fig. 2). Based on these clinical and radiographic findings, the patient was diagnosed with localized periodontitis, stage III, grade B.^[51]^

**Figure 2.**
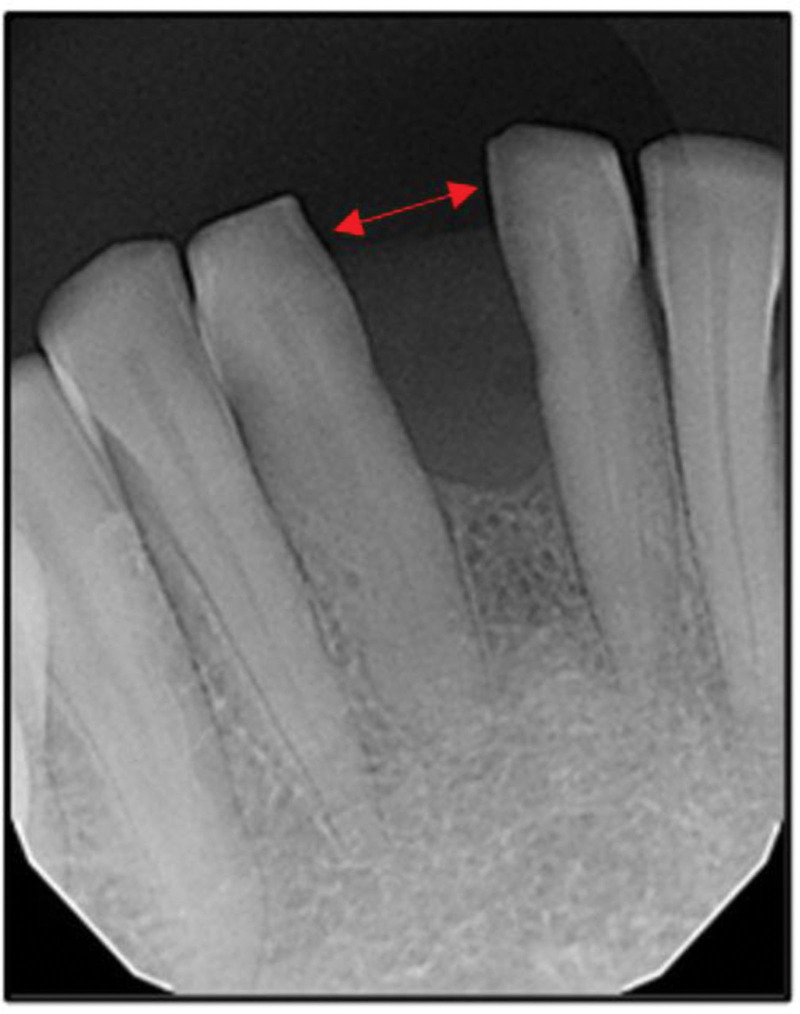
Initial preoperative periapical radiograph of the mandibular anterior region. The radiograph demonstrates horizontal bone loss and interproximal spacing (diastema) between the mandibular central incisors.

The intervention took place at the Dental Hospital of King Khalid University, involving a multidisciplinary team comprising periodontists and restorative dentists to facilitate thorough treatment planning.

## 3. Procedure

### 3.1. Presurgical preparation

Before surgical therapy, nonsurgical periodontal treatment was performed, which included patient education and motivation about comprehensive oral hygiene instructions that encompassed tooth brushing and interdental cleaning techniques, as well as scaling, root planing, and polishing (Fig. 3). A reevaluation was performed after 4 weeks to ensure the gingiva was in a healthy state before surgery.

**Figure 3.**
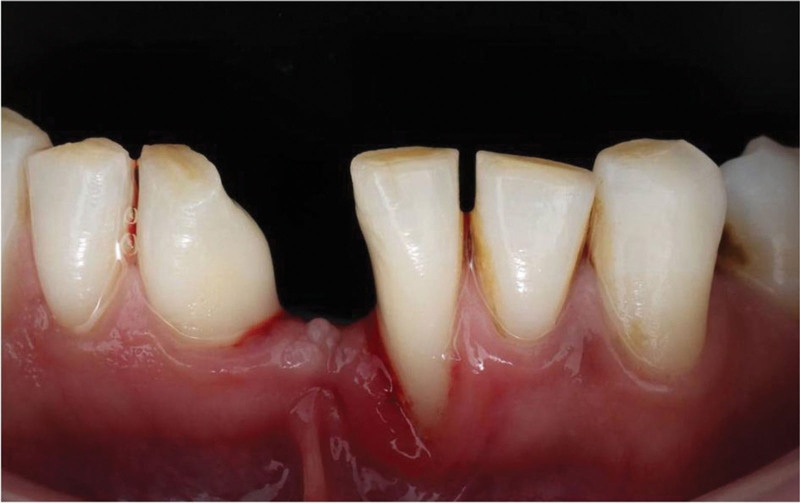
Intraoral photograph immediately after nonsurgical periodontal therapy (scaling and root planing).

The treatment plan was thoroughly explained to the patient, and informed consent was obtained. The site was treated using the Miller technique for free gingival grafting to enhance the gingival condition by increasing the width of the keratinized gingiva and achieving partial root coverage. The placement of a zirconia resin-bonded bridge followed this.

### 3.2. Surgical procedure

#### 3.2.1. Recipient site preparation

The patient was informed about the treatment plan and signed a written consent form. Under local anesthesia, the root surface was planed with a Gracey curette No. 1/2. The lower labial frenum was removed concurrently with recipient site preparation, starting with a horizontal incision made at the level of the cementoenamel junction and extending from the line angle of neighboring teeth, followed by 2 vertical incisions reaching 3 to 4 mm beyond the mucogingival junction (Fig. 4).

**Figure 4.**
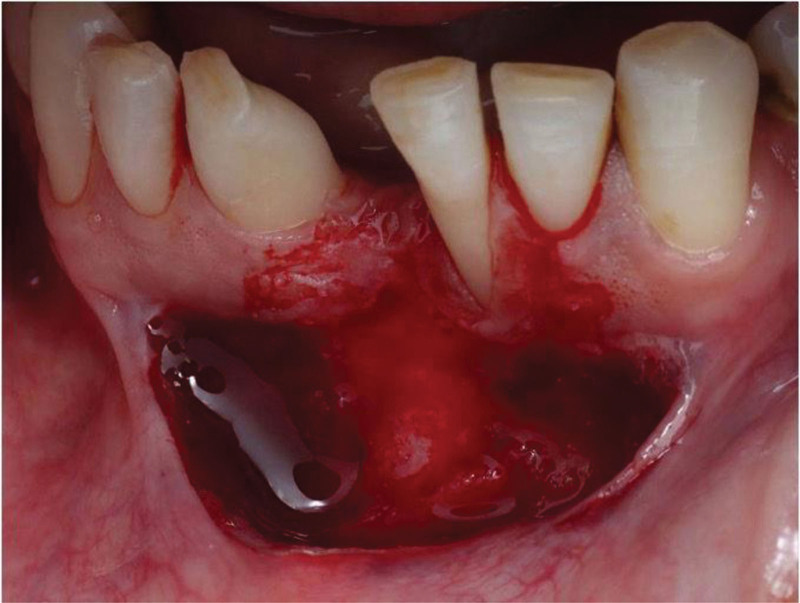
Intraoperative intraoral photograph showing the planed root surface, excision of the aberrant labial frenum, and preparation of the recipient site under local anesthesia.

A sharp dissection split-thickness flap was created, and all surfaces between the incisions and the interdental papilla were de-epithelialized using a 15C blade and a round diamond bur. The root surface, which was accessed after recipient site preparation, was planed again with a hand instrument and rinsed with saline.

#### 3.2.2. Donor site preparation

The palate thickness was measured using a periodontal probe to prepare the donor tissue for the FGG. The right palatal site between the canine and first molar was chosen 3 mm away from the gingival margin using a sterilized paper template in an inverted T-shape (Fig. 5).^[52]^

**Figure 5.**
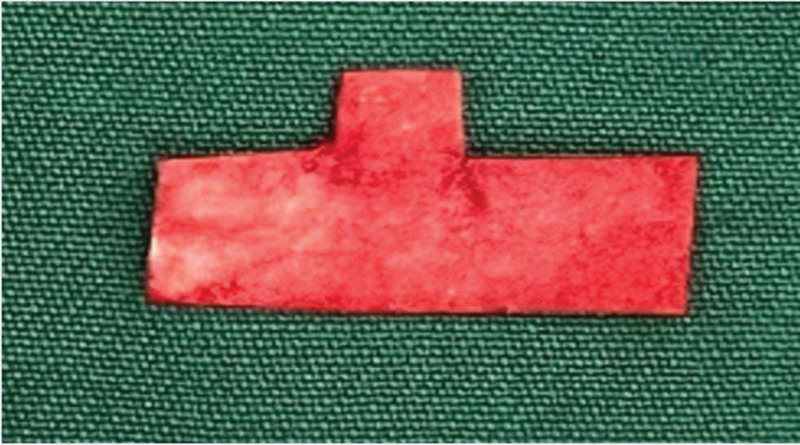
A sterilized paper template in an inverted T-shape was used to outline the dimensions of the free gingival graft and ensure precise gingival tissue harvesting from the donor site.

The graft length was around 15 mm, and the width was 6 mm, with a 3 mm extension to the interproximal papilla. The template was outlined by making an incision around it with a No. 15 scalpel blade; a bevel incision was performed to ensure the graft had an equal thickness. Tissue forceps were utilized to retract the graft during dissection, ensuring it was entirely detached. The graft was examined for any remnants of fatty or glandular tissue and its uniform thickness.

The graft was placed on the recipient site and sutured using interrupted sutures at the borders and circumferential sutures to allow for graft adaptation to the recipient site and eliminate dead space (Fig. 6).

**Figure 6.**
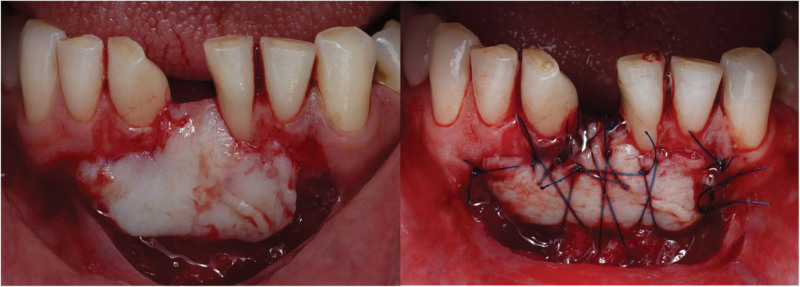
Intraoperative intraoral photograph showing the free gingival graft placed and sutured at the recipient site. Interrupted and circumferential sutures ensured adaptation and stability.

The palatal donor site wound was sealed with PeriAcryl and covered with an Essix retainer to protect the palate.

### 3.3. Postsurgical care

The patient was instructed to avoid brushing the surgical site for 3 weeks, refrain from consuming hard foods, rinse twice daily with 0.2% chlorhexidine digluconate mouthwash, take a course of antibiotics consisting of amoxicillin and ibuprofen 3 times a day for 5 days, and resume brushing gently with a soft toothbrush in the fourth week.

The suture was removed after 3 weeks, and the surgical site was rinsed with saline.

### 3.4. Postsurgical follow-up

To assess the healing, postsurgical follow-up visits were scheduled weekly during the first month and at 2 months post-surgery (Fig. 7). The healing of the FGG in the presented case provided satisfactory results, but complete root coverage was not achieved.

**Figure 7.**
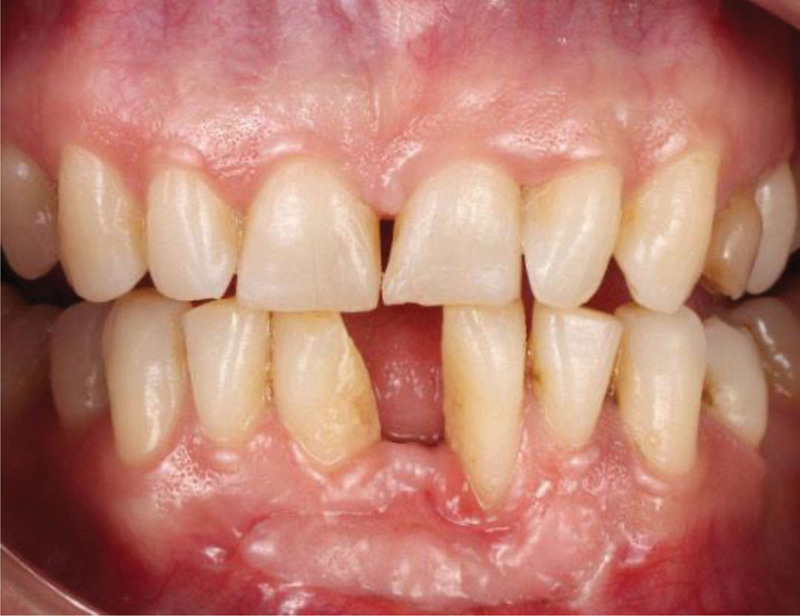
Two-month postsurgical intraoral photograph showing a completely healed, well-integrated free gingival graft and increased width of keratinized gingiva.

### 3.5. Prosthetic procedure

Two months following the periodontal surgery, the patient visited the restorative clinic for an examination.

The shade of the lower anterior teeth was recorded as (2R–2.5, Vita 3D-Master shade guide, Vita Zahnfabrik). The restorative plan, which included a resin-bonded bridge prosthesis with lingual slot preparations in teeth #31 and #41, was explained and discussed with the patient.

The final impression was taken using the “putty wash impression technique,” a light-body putty vinyl polysiloxane (Hydrorise, Zhermack). The impression was then sent to the lab for the fabrication of a zirconia resin-bonded bridge (Ceramill CAD/CAM, Amann Girrbach). During the try-in appointment, the resin-bonded bridge was evaluated for fit and proximal contact in both the cast model and the patient’s mouth. The fit of the prosthesis was evaluated by passive lingual insertion and by assessing the position of the lower border of the bridge in relation to the gingiva (Figs. 8 and 9). One shimstock paper (12 µm thick) was used to verify proper contact, and a periapical x-ray was taken.

**Figure 8.**
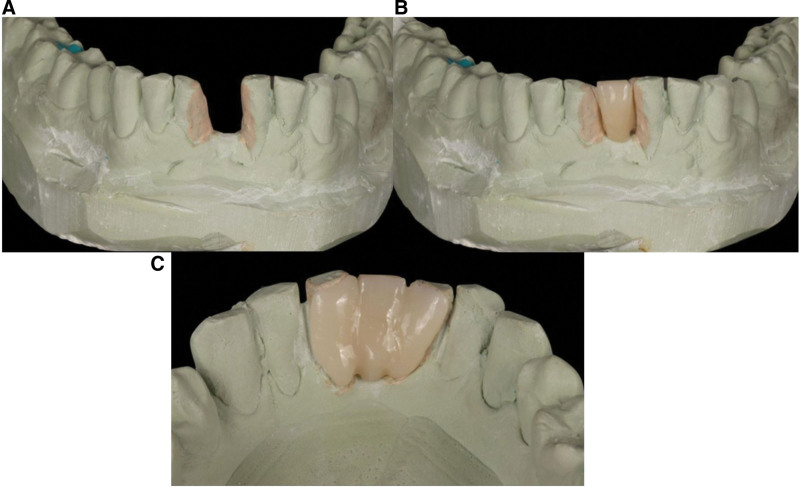
Photographs of the working cast model. (A) Illustrating the midline diastema before prosthetic rehabilitation with an RBFPD. (B) Frontal view of the diagnostic cast during the trial fitting, verifying pontic position and adaptation. (C) Lingual view of the diagnostic cast showing passive seating of the zirconia RBFPD on the lingual surfaces of teeth #31 and #41. RBFPD = resin-bonded fixed partial denture.

**Figure 9.**
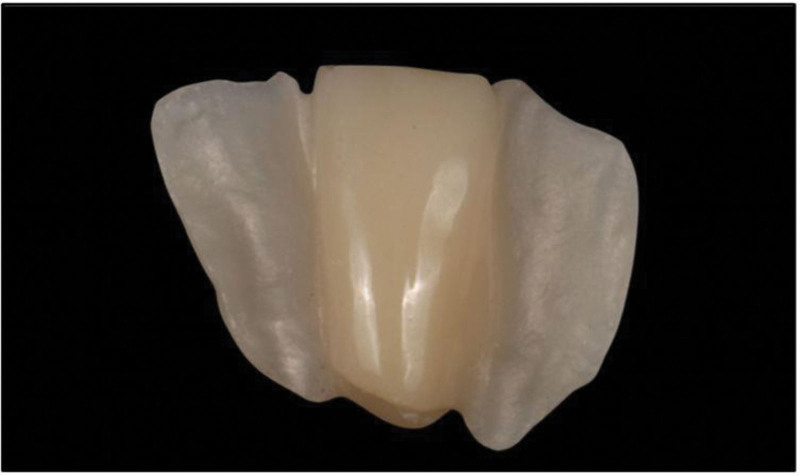
Final view of the zirconia resin-bonded fixed partial denture.

#### 3.5.1. Intaglio treatment

The resin-bonded cementation followed the “APC protocol.”^[53]^ The resin-bonded bridge’s intaglio surface was air-abraded with 50 µm aluminum oxide powder, immersed in a 90% ethanol solution, and then placed in an ultrasonic bath for 5 minutes. A 10-methacryloyloxydecyldihydrogen phosphate-containing primer was applied as a zirconia primer and allowed to dry for 1 minute. The resin-bonded bridge was cemented to the prepared tooth surface using Speed CEM Plus Resin Cement (Ivoclar Vivadent).

#### 3.5.2. Dental substrate treatment

To improve adhesive penetration and longevity, the prepared enamel surface of the resin-bonded bridge was acid-etched for 30 seconds using 37% phosphoric acid gel (Any-Etch). The etched enamel was thoroughly rinsed for 30 seconds with water and then dried with air. A bonding agent (Ardent Universal Bonding) was applied to the etched enamel using a microbrush in a rubbing motion for 20 seconds. The area was then air-dried for approximately 5 seconds to evaporate the solvent, followed by light curing for 20 seconds. Final cementation was then completed (Fig. 10).

**Figure 10.**
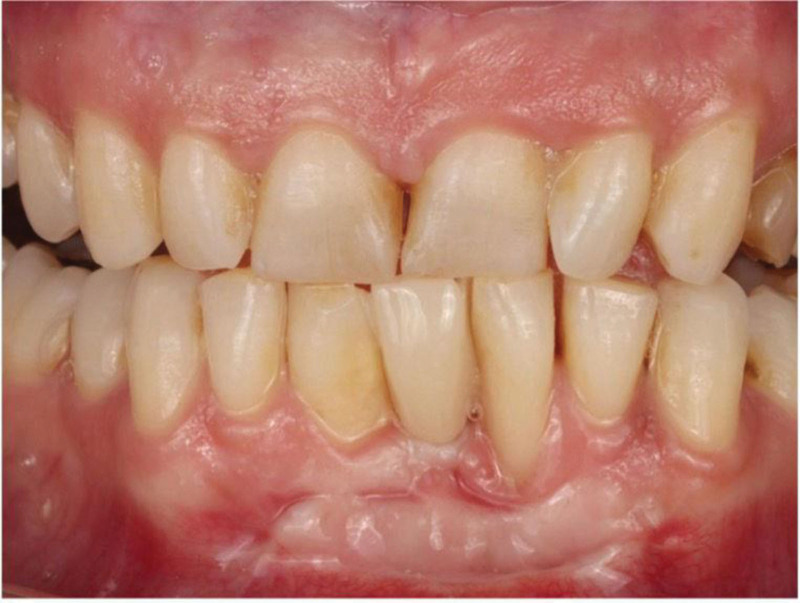
Postoperative intraoral photograph after final cementation of the zirconia resin-bonded fixed partial denture. Proper bonding, contour, and tissue adaptation are evident.

#### 3.5.3. Post-prosthetic care

The prosthesis demonstrated functionality and stability, with no debonding or marginal discoloration. The peri-prosthetic tissues showed no signs of inflammation, and there was a consistent increase in keratinized tissue width from 1 mm (pretreatment) to 5 mm (posttreatment), along with a gain of 1 to 2 mm in CAL. The midline diastema, originally measuring 6 mm, became entirely closed. The patient expressed pleasure with both the aesthetics and comfort. Clinical parameters at baseline and 4 months posttreatment are summarized in Table 1. The patient was educated on the importance of regular checkup visits and daily plaque control, which includes proper brushing and interdental cleaning techniques. This involved using a specialized form of dental floss, known as superfloss, to clean beneath the pontic of the dental prosthesis. Periodic oral hygiene instructions were reinforced at each visit. Compliance was assessed through regular plaque control records, BOP assessments, and visual inspections of prosthetic maintenance.

**Table 1:** Comparison of pre- and posttreatment clinical parameters.

Clinical parameter	Baseline (pretreatment)	4-mo posttreatment	Change (Δ)	Remarks
Diastema space (mm)	6 mm	0 mm (closed)	↓ 6 mm	Complete closure with zirconia RBFPD
Keratinized tissue width (mm)	1 mm	5 mm	↑ 4 mm	Significant tissue gain after FGG
Clinical attachment level (mm)	4–7 mm	3–5 mm	Gain ≈ 1–2 mm	Improved attachment and stability

↓ =decrease, ↑ = increase, FGG = free gingival graft, RBFPD = resin-bonded fixed partial denture.

## 4. Discussion

In this case, the diastema is the primary concern for the patient; a multidisciplinary approach, including periodontal and restorative treatments, is required.

Orthodontic therapy helps treat diastemas, producing excellent results depending on the case. Nevertheless, it is costly and time-consuming, so patients may refuse it.^[12,13]^ Mucogingival therapy, including FGGs, is performed to increase the width of keratinized gingiva, prevent gingival recession progression, and help patients maintain effective plaque control.^[23,54]^

The clinical case revealed Miller class III gingival recession associated with teeth #31 and #41, due to poor oral hygiene, inadequate width of keratinized gingiva, inadequate vestibular depth, and apparent frenum attachment. Because of these mucogingival problems, a decision was made to perform an FGG in conjunction with a frenectomy to increase the width of the keratinized gingiva, deepen the vestibule, and improve plaque control.

The decision to perform a gingival grafting procedure before placing a fixed dental prosthesis in this patient was based on the inadequate width of keratinized gingiva, gingival recession, and the increased risk of plaque accumulation and inflammation. Inadequate keratinized gingiva has been associated with compromised peri-prosthetic gingival health, leading to difficulties in plaque control, increased inflammation, and potential prosthesis failure. Therefore, gingival augmentation was performed to establish a stable periodontal foundation for long-term prosthetic success.^[55,56]^

The use of FGG may be cautiously extended to other patients with poor oral hygiene, periodontal disease, or lifestyle-related risk factors such as smoking, alcohol consumption, and recreational drug use, provided that pretreatment compliance and patient motivation are thoroughly assessed. This is particularly important when the long-term success of a prosthesis depends on the patient’s active participation in maintaining oral hygiene. However, these risk factors can significantly affect the predictability and prognosis of the graft. For instance, smoking has been shown to impair wound healing, reduce graft vascularization, and increase the likelihood of unfavorable results.^[57–61]^

Similarly, excessive alcohol consumption and substance abuse are associated with decreased patient compliance, impaired immune function, and compromised postoperative healing.^[62–66]^ FGG is contraindicated in cases of insufficient donor tissue volume, inadequate vascularity at the recipient site, and in esthetically sensitive areas due to the potential for color mismatch. It is also contraindicated in patients with systemic conditions such as uncontrolled diabetes, bleeding disorders, and those on anticoagulant therapy.^[67–69]^

While FGGs are effective in increasing the width of keratinized tissue, they carry risks and complications, including donor site morbidity, potential injury to the greater palatine artery, prolonged bleeding, postoperative pain, graft failure due to necrosis or poor integration, infection, and esthetic concerns such as color mismatch or tissue shrinkage.^[70,71]^

Alternative approaches to free gingival grafting include nonsurgical management through oral hygiene reinforcement and monitoring, prosthetic design modifications (e.g., cleansable pontics) to accommodate the decreased width of keratinized gingiva, or the use of alternative grafting materials such as allogenic or xenogenic collagen matrices in patients who are unwilling or unable to undergo autogenous grafting.^[29,72,73]^

Therefore, in such cases, strict patient selection, a behavior modification phase, and a demonstrable improvement in oral hygiene practices are essential prerequisites before considering grafting. If these conditions are met, an FGG can be successfully used to increase the width of keratinized gingiva, enhance plaque control, and improve gingival health, even in previously noncompliant individuals. However, alternative options such as soft tissue substitutes may be more appropriate in high-risk or unmotivated patients, particularly where autogenous donor site morbidity or healing compromise is a concern.

The FGG in the presented case provided satisfactory results, with an increased width of keratinized gingiva; however, complete root coverage was not achieved, consistent with Miller classification.^[74]^

In the study by Sullivan and Atkins, complete root coverage was achieved in cases of shallow and narrow recession.^[75]^ In this case, the primary purpose is to increase the width of the keratinized gingiva using an FGG and to maintain gingival health.

The gingival grafting procedure was performed by a periodontist with specialized expertise in periodontal plastic surgery. The clinician had advanced training in periodontology and had surpassed the initial learning curve, showcasing a high degree of surgical skill. This case was approached using a multidisciplinary framework, necessitating successful collaboration with an expert restorative dentist. This teamwork was crucial in ensuring that the soft tissue augmentation was effectively aligned with prosthetic planning, ultimately achieving the best results in situations involving mucogingival defects and aesthetic requirements. In this context, the effective management of soft tissues has a significant influence on the overall success of the RBFPD, improving both its functional stability and aesthetic integration.

Following the surgical procedure, restorative treatment is planned using a minimally invasive approach (RBFPD).^[76]^ This treatment was chosen for several benefits, including minimal harm to adjacent teeth and soft tissue while achieving a balance with the longevity and survival of the restoration. In contrast, placing a fixed partial denture would be a more aggressive approach, potentially leading to root canal treatment or tooth sensitivity.^[77–79]^ On the other hand, a resin-bonded bridge requires less tooth preparation.^[80]^ A fixed partial denture may compromise the periodontal ligament due to extensive restoration and compromised teeth.^[78,81]^

Due to its specific physical properties, a resin-bonded bridge with zirconia was selected. Zirconia exhibits exceptional mechanical properties, primarily attributed to a unique phenomenon known as transformation toughening.^[82–84]^ At room temperature, pure zirconia exhibits a monoclinic crystal structure. However, when heated to approximately 1170°C, it transitions to a tetragonal structure. Upon cooling, the shift from the tetragonal phase to the monoclinic phase results in a 4.5% volume increase, potentially leading to catastrophic failure.^[83–86]^ To address this issue, oxides such as calcium oxide (CaO), yttria (Y_2_O_3_), and magnesia (MgO) are used to stabilize zirconia. Among these, yttria is the most well-known dopant, as it facilitates transformation toughening, which helps prevent or at least slow the propagation of cracks and fractures.^[83,84,87–89]^

Zirconia is widely used as a dental prosthesis material due to its superior mechanical properties, biocompatibility, and aesthetics. It is commonly utilized in restorations in the form of yttria-stabilized tetragonal zirconia polycrystal.^[84,90–93]^ However, because zirconia has greater toughness and flexural strength compared with glass ceramics, larger connector dimensions are not required when fabricating zirconia prostheses.^[92]^ Moreover, zirconia demonstrates excellent biocompatibility and reduced plaque retention compared with other types of ceramics.^[94]^

In recent years, zirconia has become an increasingly popular alternative to glass-infiltrated alumina and lithium disilicate ceramics, aiming to address issues such as ceramic fractures associated with early all-ceramic RBBs.^[95]^ Five-year clinical studies found that RBBs are a reliable and practical alternative to fixed dental prostheses and implant-supported restorations in terms of survival rates.^[33,35,36,96]^ In light of the previous discussion, a resin-bonded bridge was chosen in this case as the most appropriate treatment to close the large diastema without compromising aesthetics or function.

Although the present therapy has proven beneficial, specific contraindications must be considered before expanding its application. RBBs are ineffective in patients with insufficient enamel, bruxism, or heavy occlusal forces.^[45,97]^ Therefore, proper case assessment is essential from the outset to identify situations where a resin-bonded bridge is not a feasible option. In such cases, alternative treatment options include diastema closure through orthodontics^[11]^ or composite resin restorations,^[15,16,98]^ each of which is selected based on the patient’s clinical condition and aesthetic demands.

The selection of the appropriate treatment approaches should take into account the patient’s periodontal condition, functional needs, and esthetic expectations. There are potential difficulties that may be encountered in larger clinical applications, including patient variability, technique-sensitive and donor site morbidity associated with gingival grafting, prosthesis debonding, and cosmetic differences. The result of treatment depends on the clinician’s expertise and patient compliance, as discussed in the surgical and prosthetic procedure sections of this case. This highlights the importance of restricting case selection, providing thorough patient education, and establishing established practices.

During a follow-up visit, the patient reported feeling more confident when speaking in public and less self-conscious in social situations. She explained that, before treatment, the anterior gap had caused a slight lisp, particularly when pronouncing/s/ and/z/ sounds. Following the closure of the space, she experienced improved articulation and reduced tongue protrusion during speech. These improvements contributed to her overall satisfaction with both the aesthetic and functional outcomes. She also had greater mastication efficiency, especially when chewing fibrous or chewy foods, and reported reduced strain when eating. In addition, the patient demonstrated satisfactory adherence to oral hygiene instructions and reported no discomfort or difficulty in maintaining the prosthesis during daily activities. We also monitored for signs of inflammation and prosthetic intolerance, such as tissue irritation or functional issues; none of these were observed.

The diastema closure of this complicated case was achieved successfully through a less invasive procedure, utilizing a multidisciplinary approach that included FGG and a zirconia RBFPD. The literature lacks thorough documentation of cases combining soft tissue grafting with conservative prosthetic techniques to close larger diastemas, particularly those using zirconia RBFPDs, in complex anterior mandibular areas with insufficient keratinized gingiva, despite the individual success of these treatment modalities. In a study conducted by Mangal et al,^[14]^ a metal-ceramic RBFPD was used to close a 5-mm lower midline diastema in a patient with healthy gingiva. Whereas, in this case, the periodontal condition is compromised, which necessitates surgical intervention to reduce the risk of peri-prosthetic inflammation. In addition, zirconia RBFPD was chosen instead of metal because of its superior biocompatibility, aesthetic properties, and increased survival rate.^[85,95]^

In contrast to the present case, Saygun et al^[11]^ treated a comparable clinical condition, but involved prolonged orthodontic therapy followed by composite restoration. The diastema between the lower central incisors recurred due to the patient’s noncompliance with follow-up appointments for orthodontic treatment and refusal to seek further orthodontic therapy after relapse. Lengthy orthodontic treatment requires more compliance with appointments to achieve optimal outcomes, whereas zirconia RBFPD offers a time-efficient and less invasive alternative with favorable results and enhanced durability. Composite resin was ultimately used to close the recurrent diastema area, which is known for having a lower durability and survival rate compared with zirconia RBFPD.^[33,95,96]^

The findings in this case are consistent with recent advances in prosthodontics. Mainjot^[45]^ reported a similar case of minimally invasive treatment, illustrating the feasibility of no-prep zirconia cantilever RBFPDs for single-tooth replacement. This highlights the simplicity of application and minimal intervention while preserving enamel. The current case involves the addition of FGG to increase the width of keratinized tissue, enhance stability, and improve aesthetic emergence profiles. From a biomechanical standpoint, Wang et al^[99]^ showed that zirconia frameworks distribute occlusal stresses more efficiently than other CAD/CAM materials, thereby reducing the likelihood of prosthesis failure.

The management of diastemas for aesthetic and prosthetic purposes has been addressed in various case reports. Bahrami et al^[100]^ discussed comprehensive treatment plans for congenitally missing teeth and generalized diastema, emphasizing the value of interdisciplinary collaboration. Gogoi et al^[101]^ emphasized a prosthodontic strategy to improve aesthetics in instances of midline diastema and flared incisors. Both of these studies emphasize aesthetic outcomes; however, neither included periodontal augmentation, which gives our case a distinct advantage. The concept of surface conditioning to achieve durable bonding is a critical factor, as demonstrated in the presented case. This is supported by Viana et al,^[102]^ who highlighted the importance of surface conditioning and bonding techniques in determining the clinical success of zirconia RBFPDs. Their study illustrated the benefits of enhanced surface treatment without employing the APC technique. In contrast, the presented case validated the adhesion, stability, and long-term predictability of the zirconia RBFPD by implementing the APC protocol.

All of these comparisons indicate that zirconia RBFPDs are a proven conservative treatment, and the addition of FGG enhances biological stability, providing long-term aesthetic benefits. Integrating periodontal and prosthetic approaches yielded a more comprehensive and biologically stable outcome, providing benefits that extend beyond prosthetic intervention alone. This case illustrates a unique and comprehensive approach to treating a wide mandibular midline diastema, which is associated with gingival recession and insufficient keratinized gingiva. The treatment plan included a staged protocol that involved frenectomy and FGG, followed by the placement of a time-efficient, zirconia RBFPD prosthesis, rather than relying solely on restorative or lengthy orthodontic treatment. Clinical data indicate that areas with a band of keratinized mucosa measuring ≥2 mm have considerably lower inflammation, plaque accumulation, and less gingival recession in comparison with sites with narrower keratinized areas.^[103]^ A noticeable gain in CAL was seen after the FGG treatment. This finding aligns with earlier research that has found a significant increase in attachment following mucogingival surgery.^[104,105]^ These results correspond with our case, in which pre-prosthetic soft tissue augmentation was conducted before the placement of a zirconia RBFPD in the mandibular anterior area, therefore enhancing prosthetic stability and promoting long-term soft tissue health. This approach also minimizes the risk of peri-prosthetic inflammation, plaque accumulation, and prosthesis failure by establishing adequate keratinized gingiva through the placement of FGG before prosthetic treatment, which aligns with clinical recommendations.^[23,55,56]^

From both economic and biological viewpoints, the integrated FGG and zirconia RBFPD method is significantly more cost-efficient than options such as implant support.^[45]^ It obviates the need for implant components, bone grafting, or prolonged orthodontic treatment, thereby reducing surgical morbidity, chair time, and total treatment expenses. Furthermore, by maintaining healthy neighboring teeth and attaining acceptable aesthetics through a singular soft tissue procedure and a minimally invasive prosthesis, the treatment provides a biologically conservative and economically viable solution.

For long-term surveillance, long-term follow-up is recommended to ensure that the gingival graft and prosthesis continue to function correctly. Patients should undergo regular close-up checkup visits to assess soft tissue stability and ensure the bridge is both functional and aesthetically pleasing. Each recall appointment should include an examination for symptoms of debonding, irritation, or inflammation, periodic reinforcement of oral hygiene, and visual inspections for prosthetic maintenance. This study had a short follow-up period, which is a limitation, as it may not provide a sufficiently accurate reflection of the long-term outcomes and associated issues. Further randomized controlled trials with long-term follow-up are recommended to validate the success of zirconia RBBs in managing anterior diastemas.

## 5. Conclusion

The use of a FGG is effective for increasing the width of the keratinized gingiva. However, employing this technique for root coverage is significantly more technique-sensitive. Failure to adequately address critical factors during the procedure can result in compromised outcomes.

A resin-bonded bridge is considered a preservative treatment in such cases, gaining acceptance and preference from both dentists and patients. It offers satisfactory aesthetics and functionality, making it a favorable option to close lower midline diastema compared with more invasive and time-consuming procedures, such as bone grafting or orthodontic treatment. Long-term monitoring and supportive treatment are crucial for maintaining gingival stability and ensuring the prosthesis’s functionality.

## Author contributions

**Conceptualization:** Ghadah Khaled Bahamdan, Norah Mohammed Alshehri, Faisal Hasan Alshehri.

**Data curation:** Ghadah Khaled Bahamdan, Norah Mohammed Alshehri, Faisal Hasan Alshehri.

**Funding acquisition:** Ghadah Khaled Bahamdan.

**Project administration:** Norah Mohammed Alshehri.

**Supervision:** Faisal Hasan Alshehri.

**Writing – original draft:** Ghadah Khaled Bahamdan, Norah Mohammed Alshehri, Faisal Hasan Alshehri.

**Writing – review & editing:** Ghadah Khaled Bahamdan, Norah Mohammed Alshehri, Faisal Hasan Alshehri.
